# Pulmonary hemorrhage as an early clue: an integrated clinical-imaging-genetic diagnostic insight for vascular Ehlers-Danlos syndrome

**DOI:** 10.1186/s13023-026-04327-0

**Published:** 2026-03-23

**Authors:** Yaqi Wang, Huaiya Xie, Lan Song, Kexin Xu, Junping Fan, Ping Wang, Nan Wu, Ting Zhang, Juhong Shi, Kai-Feng Xu, Xinlun Tian, Xue Zhang

**Affiliations:** 1 Department of Pulmonary and Critical Care Medicine, State Key Laboratory of Common Mechanism Research for Major Diseases, Peking Union Medical College Hospital, Chinese Academy of Medical Sciences & Peking Union Medical College, No.1 Shuaifuyuan, Dongcheng District, Beijing, 100730 China; 2 Department of Radiology, Peking Union Medical College Hospital, Chinese Academy of Medical Sciences & Peking Union Medical College, Beijing, 100730 China; 3 Department of Orthopaedic Surgery, State Key Laboratory of Complex Severe and Rare Diseases, Peking Union Medical College Hospital, Chinese Academy of Medical Sciences & Peking Union Medical College, Beijing, 100730 China; 4 State Key Laboratory of Medical Molecular Biology, Institute of Basic Medical Sciences, McKusick-Zhang Center for Genetic Medicine, Chinese Academy of Medical Sciences & Peking Union Medical College, Beijing, China

**Keywords:** Vascular Ehlers-Danlos syndrome, Pulmonary hemorrhage, Spontaneous pneumothorax, Genetic testing, Hemoptysis

## Abstract

**Background and aims:**

Vascular Ehlers-Danlos syndrome (vEDS) is a rare genetic disorder characterized by connective tissue fragility; however, respiratory manifestations such as pulmonary hemorrhage and spontaneous pneumothorax are frequently overlooked during early diagnosis. This study aimed to comprehensively characterize pulmonary features, particularly pulmonary hemorrhage, in vEDS patients and develop an integrated clinical-imaging-genetic diagnostic strategy to facilitate timely recognition and reduce diagnostic delays.

**Results:**

A single-center retrospective cohort study was conducted at Peking Union Medical College Hospital from 2016 to 2025, enrolling 32 patients with genetically confirmed vEDS. Among 32 patients, 27 (84.4%) exhibited respiratory involvement, which was defined by imaging abnormalities, with pneumothorax (50.0%) and hemoptysis (43.8%) as the most common manifestations. Patients with pulmonary hemorrhage were younger (pulmonary hemorrhage group vs. spontaneous pneumothorax and/or hemothorax group vs. Other group: 19.3 ± 4.95 vs.19.9 ± 7.98 vs. 31.7 ± 10.3, *P* = 0.002) and had fewer arterial complications (10.0% vs. 27.3% vs. 72.7%, *P* = 0.008). Chest CT revealed migratory nodules/cavities with halo signs in 40.6% cases who have normal CRP levels, indicating recurrent hemorrhage. Leveraging our summarized clinical experience regarding pulmonary hemorrhage in vEDS, median diagnostic duration shortened significantly (2.07 vs. 0.15 years, *P* = 0.001), and misdiagnosis rates dropped from 81.8% to 9.52% (*P* < 0.001), reducing unnecessary invasive procedures like bronchoscopy.

**Conclusion:**

Pulmonary hemorrhage acts as a pivotal early indicator of vEDS, corroborated by its distinctive imaging features. Our integrated clinical experience summarized from cases of pulmonary hemorrhage enhances early detection, reduces iatrogenic risks, and emphasizes the priority of genetic testing. Further multicenter prospective studies are required to validate the generalizability of this experience and optimize outcomes for this high-risk population.

**Supplementary Information:**

The online version contains supplementary material available at 10.1186/s13023-026-04327-0.

## Background

Pulmonary hemorrhage is a severe manifestation of diverse pathologic processes, ranging from localized airway bleeding to diffuse alveolar hemorrhage [1]. While autoimmune etiologies such as ANCA-associated vasculitis and Goodpasture syndrome are well-recognized culprits, the diagnostic landscape is further complicated by a heterogeneous group of rare disorders [2–6]. These conditions can mimic immune etiologies, creating a complex diagnostic dilemma that requires a meticulous and multidisciplinary approach.

Among these rare etiologies, vascular EDS (vEDS), formerly known as type IV EDS, is widely recognized for its severe manifestations and poor prognosis [7–11]. Caused by pathogenic variants in the *COL3A1* gene encoding type III collagen, vEDS is characterized by catastrophic arterial and organ rupture due to defective connective tissue integrity, with a median life expectancy of 48–51 years [12]. Ongoing investigations into medication and genotype-informed management strategies hold the potential to mitigate the risks of these potentially lethal complications, but timely and accurate diagnosis remains a critical challenge. Although spontaneous pneumothorax has been incorporated into the diagnostic criteria, other respiratory features, including fibrous nodules, bullous emphysema, cystic lung lesions, and pulmonary hemorrhage, remained poorly characterized [13–17]. While pulmonary hemorrhage has been sporadically reported in young patients with vEDS, its prevalence, radiographic characteristics, and potential utility as an early diagnostic indicator remain under-investigated [14–16, 18, 19].

To address this gap, we conducted a single-center retrospective cohort study to comprehensively characterize the respiratory manifestations, particularly pulmonary hemorrhage, in patients with vEDS. In 2022, we identified clinical and imaging features of intrapulmonary hemorrhage, which, combined with genetic testing, were expected to serve as an early diagnostic clue for vEDS. This insight aims to address the current diagnostic challenge in vEDS cases presenting with pulmonary hemorrhage, expedite diagnosis and treatment, and ultimately improve clinical outcomes.

## Materials and methods

### Study cohort and patients

This retrospective study enrolled patients with vEDS presented at Peking Union Medical College Hospital (PUMCH) from July 2016 to October 2025. The diagnosis of vEDS was established according to the 2017 international classification of the Ehlers-Danlos syndromes, including: (1) patients with gene mutation of *COL3A1* (most cases) or *COL1A1* (rare), and (2) patients met one of the following minimal criteria: (a) a family history of vEDS; (b) arterial rupture or dissection in individuals less than 40 years of age; (c) unexplained sigmoid colon rupture; or (d) spontaneous pneumothorax in the presence of other features consistent with vEDS [17]. This study was approved by the Institutional Review Board of PUMCH (I-25PJ1533). As a retrospective study, the requirement for informed consent was waived.

### Data collection

All patients with genetically confirmed vEDS were identified and reviewed by searching for “EDS” or “Ehlers-Danlos Syndrome” in their medical records via the Big Data Query and Analysis System of PUMCH. Clinical data were obtained from the electronic medical record system, including: (1) demographics (age of first visit and sex), clinical manifestations (respiratory symptoms, artery complications, solid or hollow organ ruptures, and common features of EDS) and time interval from first visit at PUMCH to diagnosis; (2) genetic variants in *COL3A1*; (3) laboratory results: white blood cell (WBC), neutrophil count and percentage, C reactive protein (CRP), erythrocyte sedimentation (ESR), antinuclear antibody (ANA), and anti-neutrophil cytoplasmic antibody (ANCA); (4) findings on chest CT scans; (5) procedures, such as bronchoscopy, video-assisted thoracic surgery (VATS) biopsy, bullectomy, and closed chest drainage; and (6) treatment administered prior to diagnosis, such as antibiotics, anti-tuberculous and corticosteroid. Artery complications included aneurysm, arterial rupture and arterial dissection. The common features of EDS were defined as joint hypermobility, thin and translucent skin, skin hyperextensibility, and ecchymosis.

### Chest CT scan analysis

All chest CT scans were assessed independently by two experienced respiratory physicians (H.X. and T.Z.). In case of disagreement, the final decision was made by a third respiratory physician (X.T.). All abnormal chest imaging findings were finally confirmed by a radiologist (L.S.). The types and definitions of chest CT abnormalities are summarized in Supplementary Table S1 [20].

Patients with vEDS were further divided into three subgroups according to a combination of clinical manifestations, chest imaging features and pulmonary histopathological results (if available): the pulmonary hemorrhage (PH) group, the spontaneous pneumothorax and/or hemothorax (P/HTX) group, and other manifestations (OTHER) group. Pulmonary hemorrhage (PH) was defined by meeting any of the following criteria: (1) lung pathology showing acute or chronic hemorrhage (aggregation of hemosiderin-laden macrophages) and hematoma formation; (2) serial aliquots of bronchoalveolar lavage revealing an increasingly hemorrhagic or persistently bloody return; (3) hemoptysis with at least one of the following imaging findings: wandering lesions (nodules, cavities, patches), halo sign, alveolar filling ground glass opacities (GGO)/consolidation. The PH Group included patients who met the diagnostic criteria for spontaneous pulmonary hemorrhage, either in isolation or in combination with other manifestations. The P/HTX Group comprised patients who presented with pneumothorax or hemothorax in the absence of pulmonary hemorrhage, with or without additional respiratory manifestations. The OTHER group encompassed the remaining vEDS patients who did not meet the diagnostic criteria for the PH group or P/HTX group.

### Statistical analysis

Respiratory system involvement was defined as abnormal manifestations on chest CT, with or without respiratory system symptoms. While spontaneous pneumothorax in vEDS has been well-recognized in clinical practice, pulmonary hemorrhage as an early respiratory manifestation remains underrecognized, and thus we performed subgroup statistical comparisons to characterize its clinical and imaging features. Diagnostic duration was defined as the time interval from the patient’s initial visit at PUMCH to the establishment of diagnosis. Continuous variables were reported as mean ± standard deviation (SD) or median with interquartile range [IQR], based on the normality of distribution. Categorical variables were described as the counts and percentages. The chi-square test, Fisher’s exact test, ANOVA, and Kruskal-Wallis rank sum test was used to compare clinical characteristics between different groups. All statistical analyses were conducted using R (version 4.3.2, R Foundation for Statistical Computing, Vienna, Austria). All statistical tests were two-tailed, and a *P* < 0.05 was considered statistically significant.

## Results

### Clinical characteristics of vEDS

**Fig. 1:**
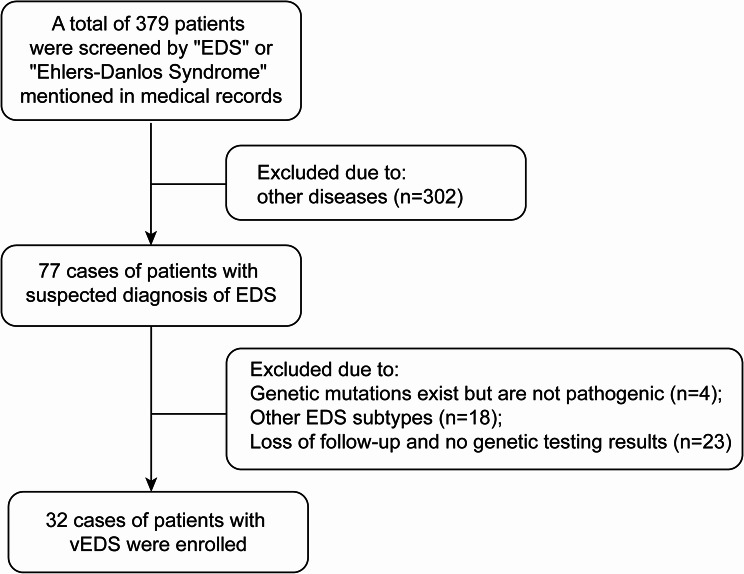
The search and screening patients with vascular Ehlers-Danlos syndrome. EDS: Ehlers-Danlos syndrome; vEDS: vascular Ehlers-Danlos syndrome

A total of 32 patients with vEDS were reviewed and identified (Fig. 1). Common signs of vEDS were observed in 20 patients. Fourteen patients had positive family histories. All 32 patients carried disease-causing variants in *COL3A1*, identified through exome sequencing (Supplementary Tables S2).

Twenty-seven patients with vEDS exhibited respiratory system involvement, among which pneumothorax (50.0%) and hemoptysis (43.8%) were the most common respiratory symptoms. The average age of PH group and P/HTX group was lower than other group, although the difference did not reach statistical significance (PH group vs. P/HTX vs. OTHER group: 19.3 ± 4.95 vs.19.9 ± 7.98 vs. 31.7 ± 10.3, *P* = 0.002). No significant differences were observed in diagnostic duration between two groups. The incidence of extrapulmonary vascular complications was significantly lower in PH group and P/HTX group (10.0% vs. 27.3% vs. 72.7%, *P* = 0.008), although this difference may be confounded by age, as vascular complications typically occur later in life, indicating that respiratory symptoms may serve as an early indicator of vEDS [21]. There was no significant difference in the prevalence of common features of EDS between groups.

Patients with vEDS did not exhibit evidence of an overt inflammatory response or infection. The median values of WBC and neutrophils (count and percentage) were in the normal range. CRP and ESR were not significantly elevated, indicating a non-inflammatory pathological mechanism underlying respiratory involvement in vEDS. Among patients who underwent autoantibody testing (ANA and ANCA), only one patient tested positive for ANA. The proportion of patients in the PH group who underwent bronchoscopy with bronchoalveolar lavage for pathogen screening was significantly higher (70.0% vs. 0.00% vs. 0.00%, *P* < 0.0001), and no pathogens were detected (Table 1).

Patients with PH or P/HTX were more likely to undergo additional invasive procedures, with the proportion of those receiving such interventions higher (80.0% vs. 63.6% vs. 0.00%, *P* < 0.001); notably, 4 patients in PH group underwent percutaneous, transbronchial, or surgical lung biopsy solely for diagnostic purposes. Seven patients underwent surgical treatment for pneumothorax, with 3 receiving lung biopsies during the procedure (Table 2, Supplementary Tables S3).

The rate of suspected infection leading to inappropriate treatment in patients with PH was significantly higher (*P* < 0.001). Specifically, among all patients, 8 patients received antibiotics for suspected infection, 2 patients received quinolone and metronidazole for suspected parasitic infection, and 2 patients received diagnostic anti-tuberculosis therapy. Three patients were misdiagnosed with vasculitis and treated with corticosteroid. None of these treatments were effective. During follow-up, only one patient developed an arterial complication, which was a right subclavian artery aneurysm occurred after the diagnosis was established (Table 1).

**Table 1:** Clinical characteristics of patients with vEDS presenting pulmonary involvement

	PH group (*n* = 10)	P/HTX group (*n* = 11)	Other group (*n* = 11)	*P*
**Age of first visit, years**	19.3 ± 4.95	19.9 ± 7.98	31.7 ± 10.3	0.002
**Male**,** n (%)**	6 (60.0)	9 (81.8)	9 (81.8)	0.42
**Artery complication**,** n (%)**	1 (10.0)	3 (27.3)	8 (72.7)	0.008
**Visceral organ rupture**,** n (%)**	0 (0.00)	1 (9.09)	0 (0.00)	0.37
**Common features**	7 (70.0)	7 (63.6)	6 (54.6)	0.76
Skin hyperextensibility, n (%)	1 (10.0)	1 (9.09)	2 (18.2)	0.78
Joint hypermobility, n (%)	5 (50.00)	5 (45.5)	5 (45.5)	0.97
Thin and translucent skin, n (%)	2 (20.0)	1 (9.09)	3 (27.3)	0.55
Ecchymosis, n (%)	5 (50.0)	5 (45.5)	3 (27.3)	0.53
**Laboratory test**				
WBC, ×10^9^/L	9.06 [5.92, 10.0]	6.08 [5.52, 6.81]	6.19 [5.49, 9.01]	0.71
Neutrophil, ×10^9^/L	4.26 [3.52, 5.86]	4.09 [3.43, 4.50]	3.96 [3.63, 5.91]	0.86
Neutrophil, %	55.5 ± 6.26	63.6 ± 10.4	56.8 ± 14.2	0.26
C reactive protein, mg/L	2.48 [0.97, 3.88]	3.88 [1.35, 7.57]	0.96 [0.70, 6.27]	0.91
ESR, mm/h	5.50 [2.75, 6.25]	11.0 [5.00, 12.0]	6.50 [5.75, 7.25]	0.49
Positive ANA, %	0 (0.00)	0 (0.00)	1 (1/5, 20.0)	0.17
**Misdiagnose (%)**	7 (70.0)	1 (9.09)	3 (27.3)	0.01
Vasculitis (%)	1 (10.0)	0 (0.00)	2 (18.2)	0.34
Infection (%)	7 (70.0)	1 (9.09)	0 (0.00)	< 0.001
Diagnostic duration*,** years**	0.50 [0.12, 2.77]	0.19 [0.13, 0.41]	0.21 [0.14, 0.36]	0.65
**Positive family history**,** n (%)**	5 (50.0)	5 (45.5)	4 (36.4)	0.81

*Diagnostic duration was defined as the time interval from first visit at PUMCH to diagnosis

**Table 2:** Procedures and treatments of patients with vEDS

	PH group (*n* = 10)	P/HTX group (*n* = 11)	Other group (*n* = 11)	*P*
**Invasive procedure**	8 (80.0)	7 (63.6)	0 (0.00)	< 0.001
Bronchoscopy (%)	7 (70.0)	0 (0.00)	0 (0.00)	< 0.001
Lung biopsy	4 (40.0)	3 (27.3)	0 (0.00)	0.075
Percutaneous (%)	2 (20.0)	0 (0.00)	0 (0.00)	0.14
Transbronchial (%)	2 (20.0)	0 (0.00)	0 (0.00)	0.10
Surgical (%)	2 (20.0)	3 (27.3)	0 (0.00)	0.19
Surgery (%)	4 (40.0)	7 (63.6)	1 (9.09)	0.03
**Treatment**				
Antibiotic (%)	7 (70.0)	1 (9.09)	0 (0.00)	< 0.001
Corticosteroid (%)	1 (10.0)	0 (0.00)	3 (27.3)	0.15
Anti-tuberculous (%)	2 (20.0)	1 (9.09)	0 (0.00)	0.29

### Chest CT findings

**Fig. 2:**
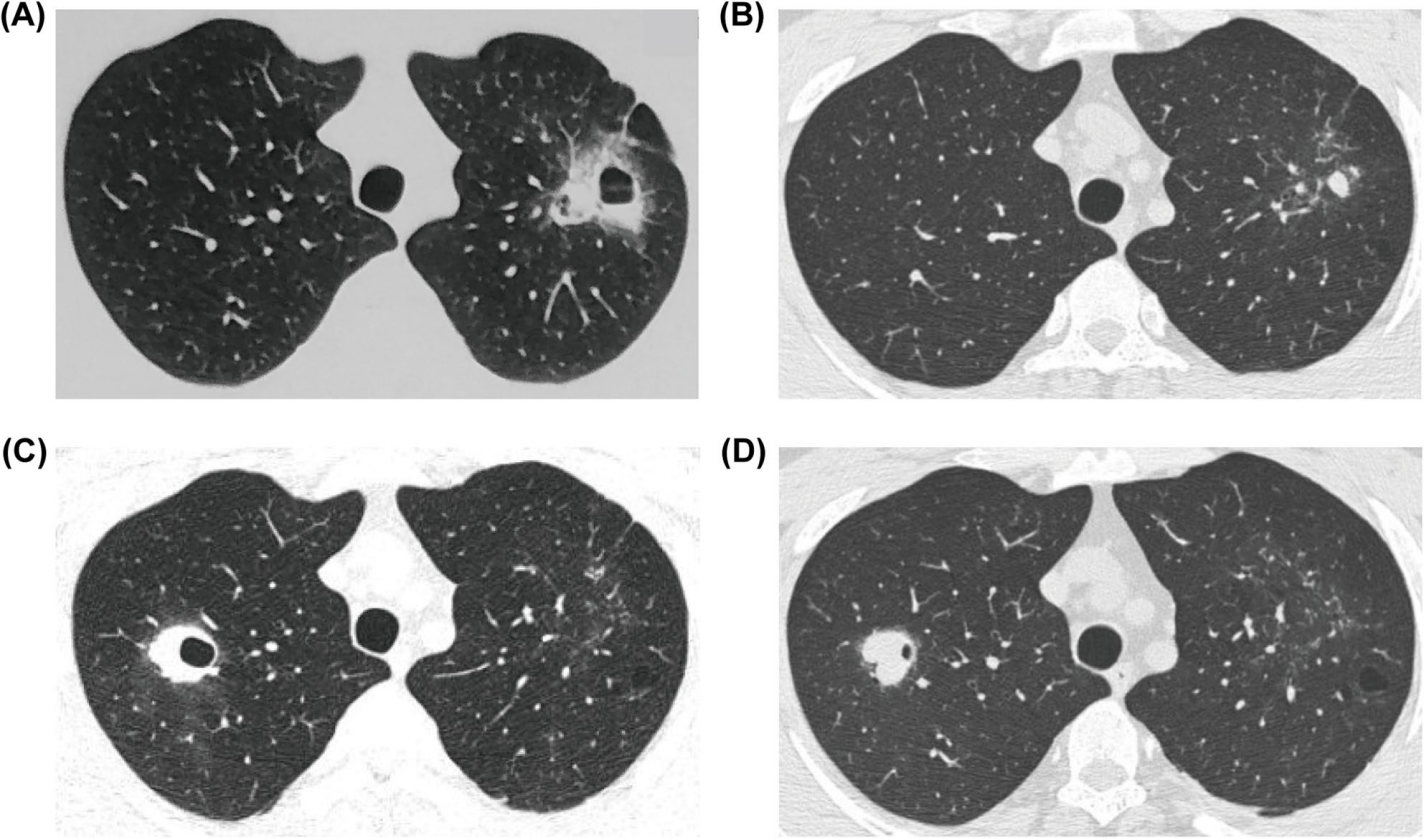
Representative migratory lesions of vascular Ehlers-Danlos syndrome. A 22-year-old man presented with waxing and waning lesions in the lung. (**A**, **B**) Chest CT on admission revealed cavity with halo sign in left upper lobe. (**C**, **D**) Chest CT taken four months later showed resolution of the prior lesion; however, a new nodule and cavity with a halo sign had developed in the right upper lobe

The chest CT abnormalities of patients with vEDS were summarized in Table 2. Spontaneous pneumothorax/hemothorax (18, 56.3%) and pulmonary bulla/emphysema (17, 53.1%) were the most common findings. Nodules were found in 14 patients (43.8%), of which 11 (78.6%) with cavitation, and 10 (71.4%) with halo sign, a radiological feature suggestive of intrapulmonary hemorrhage. Notably, the inner walls of the cavity were generally smooth. A total of 13 patients (40.6%) had migratory lesions, including nodules, cavities, patchy GGO or consolidations, that waxed and waned spontaneously during the course of the disease (Table 3; Fig. 2, Supplementary Fig. S1).

**Table 3:** Chest CT findings of patients with vEDS

CT Findings, *n* (%)	Patients with vEDS (*n* = 32)
Lung bulla /emphysema	17 (53.1)
Spontaneous pneumothorax/hemothorax	18 (56.3)
Migratory lesions	13 (40.6)
Ground-glass opacities	11 (34.4)
Nodules*	14 (43.8)
Nodules with cavities	11 (34.4)
Halo sign	10 (31.3)
Other findings	
Linear opacities	16 (50.0)
Fibrous nodules	15 (46.9)
Bronchiectasis/bronchiolectasis	7 (21.9)
Calcified nodules	3 (9.38)
Subpleural lines	1 (3.12)

*Fibrous nodules and calcified nodules were excluded

### Clinical features of pulmonary hemorrhage in vEDS

**Fig. 3:**
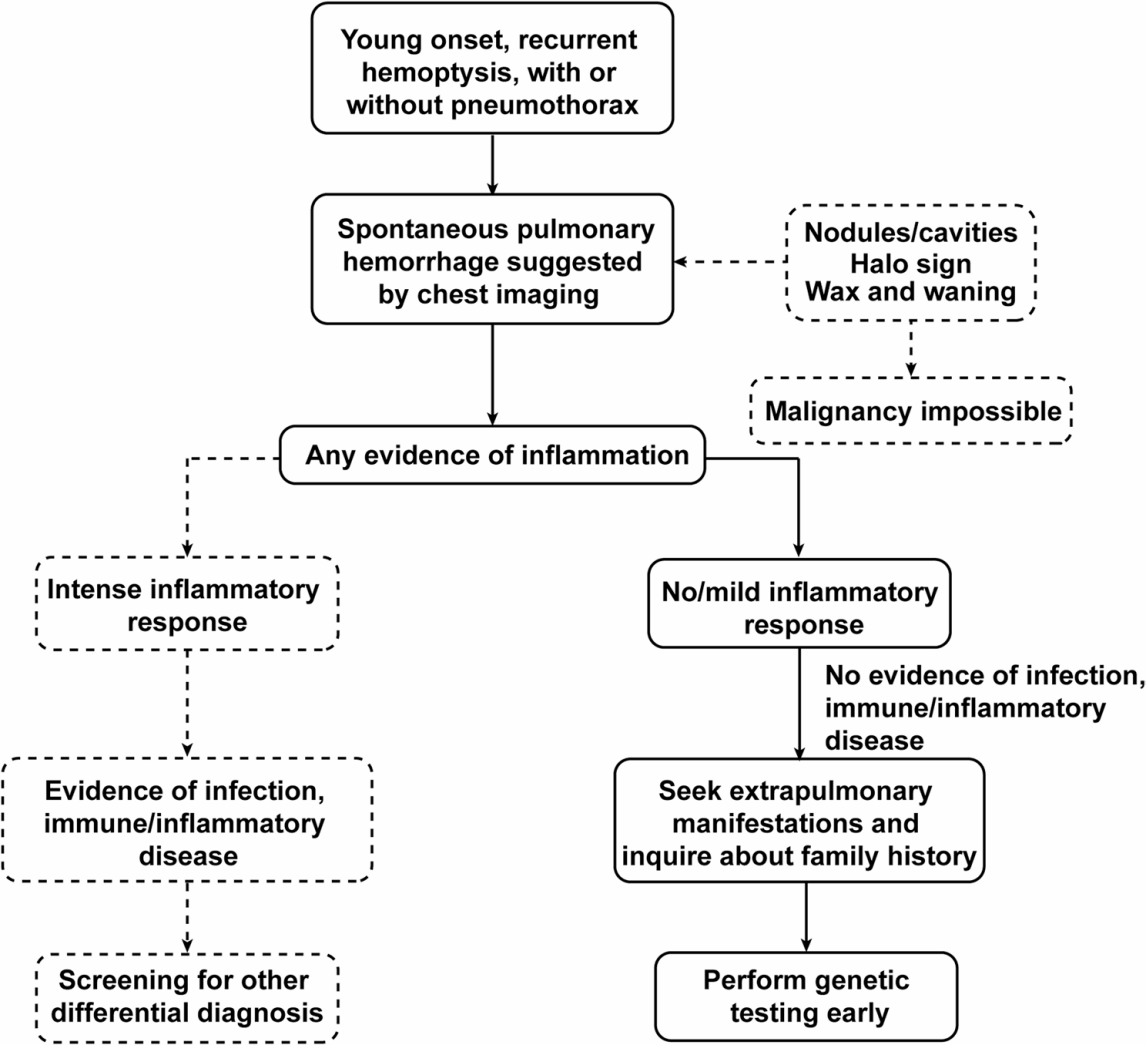
Experience-based guidance for recognizing vEDS with respiratory manifestations, emphasizing pulmonary hemorrhage

Based on the findings of our cohort, we summarized the clinical-imaging-genetic features of pulmonary hemorrhage in vEDS. These features are intended to serve as clues for the early recognition and diagnosis of vEDS presenting with initial respiratory manifestations, thereby supplementing the current diagnostic criteria: (1) young patients with recurrent hemoptysis, with or without history of pneumothorax/hemothorax; (2) chest imaging findings suggestive of recurrent intrapulmonary hemorrhage (nodules or cavities with halo sign that waning and waxing); (3) absence of evidence for inflammation or infection, as indicated by normal WBC, neutrophil, CRP, ESR, as well as negative pathogen screening; and (4) low clinical suspicion of malignancy, supported by radiological evidence showing self-resolving lesions. In patients presenting with the above characteristics, the skin and joint should be carefully examined, and a full family history should be collected. However, the absence of joint hypermobility, skin hyperextensibility, or a family history does not exclude the possibility of vEDS. Once vEDS is suspected, genetic testing should be proactively performed, while biopsy, whether transbronchial, percutaneous, or surgical, should be deferred until the diagnosis of vEDS is excluded (Fig. 3).

**Fig. 4:**
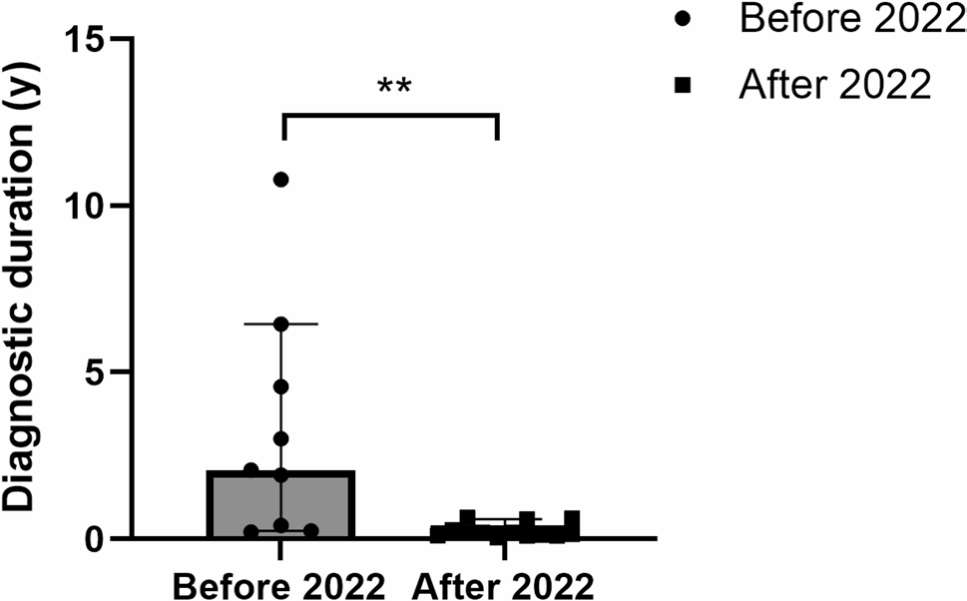
Diagnostic duration for patients with vEDS before and after empirical summary. Before 2022: initial visit before 2022; after 2022: initial visit after 2022. ^**^P < 0.01

Since 2022, we began focusing on and summarizing the characteristics of pulmonary hemorrhage in vEDS, and on this basis, implemented the experience-based diagnostic insights for vEDS with respiratory manifestations as illustrated in Fig. 3. Following this implementation, the diagnostic duration of vEDS has been shortened significantly (initial visit before 2022 vs. initial visit after 2022: 2.07 [IQR 0.41, 4.57] vs. 0.15 [0.09, 0.21], *P* = 0.001) (Fig. 4). During the same period, the misdiagnosis rate also decreased significantly (81.8% vs. 9.52%, *P* < 0.001), and the proportion of patients undergoing bronchoscopy has decreased as well, although this difference did not reach statistical significance (45.5% vs. 9.52%, *P* = 0.06) (Table 4, Supplementary Table S4-5).

**Table 4:** Clinical features of patients with vEDS who visited the hospital before and after 2022

	First visit before 2022 (*n* = 11)	First visit after 2022 (*n* = 21)	*P*
Misdiagnose, *n* (%)	9 (81.8)	2 (9.52)	< 0.001
Bronchoscopy, n (%)	5 (45.5)	2 (9.52)	0.06
Lung biopsy for diagnosis, n (%)	3 (27.3)	1 (4.76)	0.21
Diagnostic duration^*^, years	2.07 [0.41, 4.57]	0.15 [0.09, 0.21]	**0.001**

*Diagnostic duration was defined as the time interval from first visit at PUMCH to diagnosis

## Discussion

This study comprehensively characterized the pulmonary manifestations of vEDS and identified pulmonary hemorrhage as a frequently underrecognized feature, manifesting as early-onset migratory lesions on chest CT without inflammatory findings. We therefore proposed a novel clinical insight that integrates pulmonary hemorrhage as a key diagnostic indicator and prioritizes genetic testing over invasive procedures. This empirical summary has been shown to effectively shorten diagnostic delays, reduce misdiagnosis rates, and limit the application of high-risk invasive interventions. These findings highlight that leveraging pulmonary hemorrhage as a core clue, combined with prioritization of genetic testing, enables timely and accurate diagnosis of vEDS with pulmonary involvement, while lowering iatrogenic risks. With the rapid advancement of clinical management strategies, this practice-based approach holds significant potential to mitigate life-threatening complications and ultimately improve patient outcomes.

Vascular EDS is frequently diagnosed following the sudden onset of arterial or organ ruptures, which can be fatal. While spontaneous pneumothorax is a well-documented major feature, other respiratory manifestations of vEDS have only been described in case reports and remain largely underrecognized [22, 23]. However, our study demonstrated that respiratory involvement was not rare in vEDS patients. Importantly, hemoptysis (43.8%) was the second most prevalent respiratory symptom, following spontaneous pneumothorax, and this rate was significantly higher than previously reported [21, 24]. This finding also suggested that vEDS may be an underlying cause of hemoptysis in a subset of patients, a possibility that may have been previously overlooked.

Herein, we present a comprehensive characterization of chest imaging findings, especially those regarding pulmonary hemorrhage, in vEDS. Consistent with the previous studies, pneumothorax/hemothorax and emphysema/bullae were the most common respiratory complications and CT findings in our cohort [23–25]. Reports of intrapulmonary hemorrhage in vEDS are limited to isolated cases, without detailed imaging features or data on prevalence [14–16, 19]. In our cohort, imaging features suggestive of pulmonary hemorrhage were highly prevalent, including a higher rate of cavitated nodules than in previous reports, with 31.3% of patients showing nodules with halo signs and 40.6% exhibiting waxing and waning features of pulmonary lesions on serial chest CT scans [24]. The presence of both acute and chronic lesions indicated the clinical manifestations of the recurrent episodes of spontaneous injuries of the fragile lungs, which may be one of the clinical clues of vEDS.

**Fig. 5:**
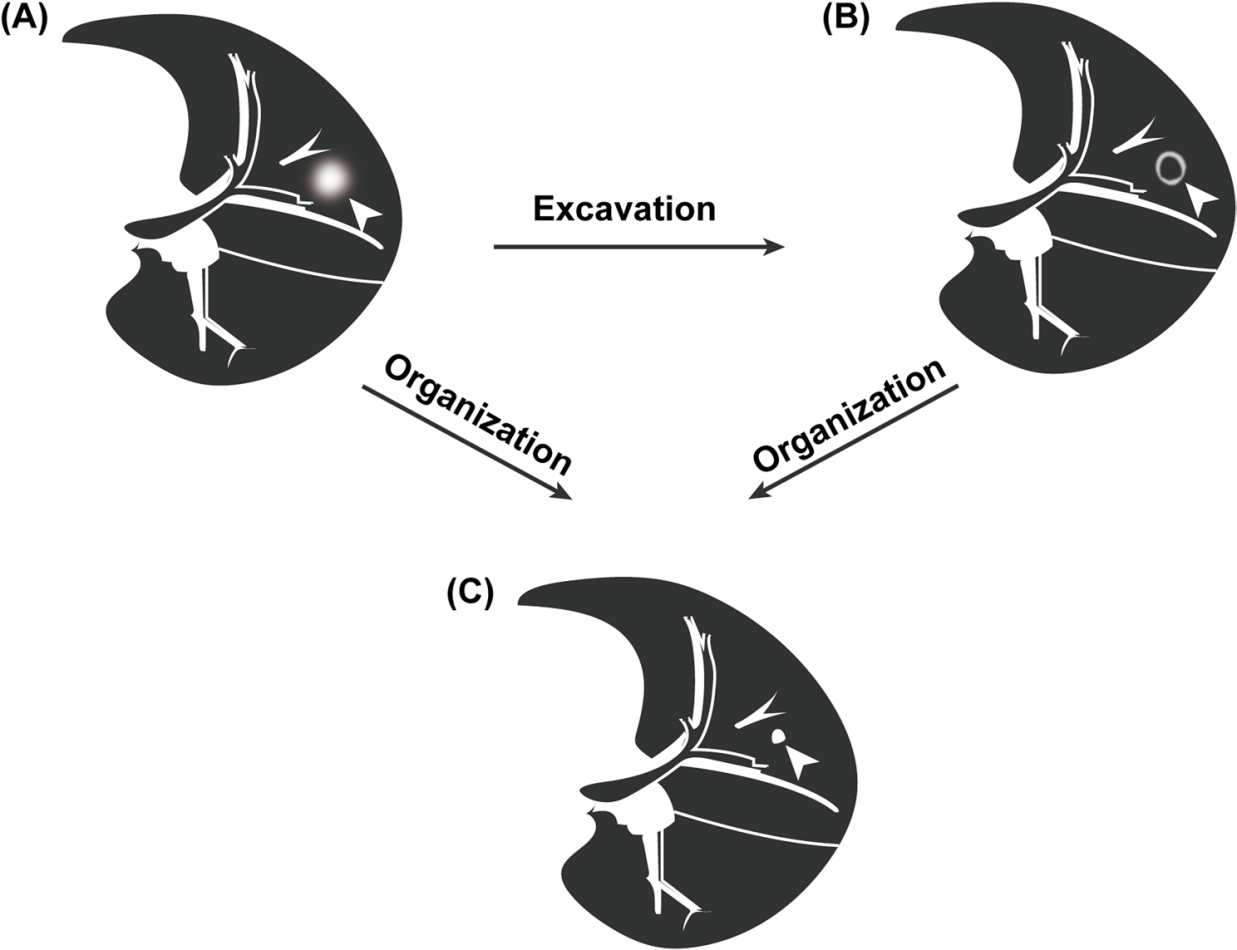
Schematic illustration of the evolution of spontaneous intrapulmonary hemorrhage in patients with vEDS. (**A**) Acute hematoma is formed by spontaneous rupture of pulmonary blood vessels. (**B**) Cavity is formed after excavation of the acute hematoma. (**C**) Fibrous nodule is formed after the organization and fibrosis of the hematoma

Our finding that intrapulmonary hemorrhage can serve as an indicative imaging feature of vEDS is consistent with its underlying pathophysiological mechanisms. Pathogenic variants in *COL3A1*, particularly those leading to glycine substitutions disrupting the normal helical structure of collagen, results in fragility of the vascular and alveolar walls [26, 27]. Kawabata et al. [25] showed that hematoma, caused by spontaneous laceration of lung or vascular disruption, is a key pathological feature of vEDS. Following hematoma formation and excavation, the self-repairing mechanism is initiated, characterized by tissue organization and fibrosis. This pathological sequence—disruption, hemorrhage, hematoma formation and following evolution—correlates with the radiological findings of nodules with halo sign, cavity formation, and spontaneous shrinkage into a small fibrous nodule (Fig. 5) [24, 28]. The recurrent injury process, which includes laceration, hemorrhage, hematoma formation, along with the subsequent repair process involving organization, fibrosis and hematoma absorption, explains the waning and waxing features of lesions on chest CT. On the other hand, minor spontaneous injuries to the alveolar wall without vascular or capillary involvement leads to the formation of emphysema and blebs.

Patients with pulmonary hemorrhage experienced higher misdiagnosis rates of infection, leading to inappropriate diagnostic procedures and administration of unnecessary or potentially harmful treatments. A substantial portion of the patients in our cohort underwent invasive procedures before 2022, including bronchoscopy and lung biopsy. However, these procedures did not yield a definite diagnosis beyond confirming pulmonary hemorrhage, which could be recognized based on the characteristic symptoms and radiological features [29]. Genetic test is a crucial and noninvasive tool for establishing a definitive diagnosis of vEDS and was performed in all of the patients in our study. Given the high risk of unexpected and potentially fatal complications from invasive procedures in patients with vEDS, which are attributable to the fragile connective tissues, genetic test should be prioritized over biopsy and other diagnostic methods in all patients with suspected vEDS [30, 31].

Respiratory complications often occur prior to the onset of vascular events, but clinical attention primarily focused on pneumothorax/hemothorax [21]. In our cohort, ten patients presented with hemoptysis as the initial clinical manifestation. Among these 10 patients, no patient developed aneurysms during follow-up. The younger onset of hemoptysis and low incidence of extrapulmonary complications suggested that pulmonary hemorrhage may be an underrecognized early clinical manifestation in a subset of patients with vEDS.

Preventive pharmacotherapy and genotype-informed management plans mitigate the risks of vascular events; therefore, early diagnosis is crucial for extending lifespan and improve quality of life [32]. Accurate identification of vEDS based on clinical manifestation remains a fundamental step in diagnosis. The current primary diagnostic criteria for vEDS predominantly focus on severe complications, such as vascular events and intestinal rupture, which are often fatal once they occur. Given the critical nature of these complications, there is an urgent need to identify other clinical clues that can signal the presence of vEDS at an earlier stage. With the gradual deepening of understanding of vEDS, the increasing availability of genetic testing, and clinicians’ proactive efforts in identifying the etiology, the diagnostic rate of vEDS has been significantly improved. Our study suggested that hemoptysis, particularly in the absence of an obvious inflammatory response, should prompt consideration of vEDS, supplementing the current diagnostic process. By applying the empirical insights summarized from pulmonary hemorrhage characteristics in vEDS, we have achieved significant improvements in diagnostic accuracy, shortened the diagnostic duration, and reduced the use of diagnostic biopsies (Fig. 4).

There are several limitations of our study. First, as a single-center retrospective study and given the rarity of vEDS, the sample size was limited, which may increase the risk of selection bias and limit the generalizability of the conclusions to other populations and clinical settings. However, two previously published studies have reported similar findings, specifically that manifestations of pulmonary hemorrhage, including hemoptysis and characteristic imaging features, may serve as early indicators of vEDS [18, 19]. To a certain extent, these findings supported the generalizability of our observations. Second, as a rare disease, the relatively small size of our cohort led to low statistical power, which may increase the risk of Type II errors. To minimize this bias, we included all consecutive patient with patients with vEDS who visited PUMCH between 2016 and 2025, identified through the Big Data Query and Analysis System. Third, the follow-up duration was relatively short, limiting our ability to fully assess longitudinal outcomes and the relationship between respiratory features and prognosis. Fourth, these early observations and empirical summaries have not been validated in prospective cohorts and do not constitute a mature diagnostic framework. Future multicenter prospective cohort studies, using pulmonary hemorrhage as an inclusion criterion, are needed to validate the sensitivity and specificity of this diagnostic approach, as well as to investigate the longitudinal changes in pulmonary and extrapulmonary outcomes of patients with vEDS.

In conclusion, recurrent hemoptysis and hematoma formation without elevated inflammatory markers in vEDS often present as nodules or cavities with halo signs characterized by waxing and waning features on serial chest CT scan. These findings should prompt clinical suspicion of vEDS, particularly in patients with early-onset symptoms and no definitive inflammatory evidence. Our center’s summarized empirical experience, with pulmonary hemorrhage as a key diagnostic clue, contributes to early recognition and diagnosis of vEDS with initial respiratory manifestations. Multicenter prospective cohort studies are required to validate and improve the clinical applicability and generalizability of this experience-based approach.

## Supplementary Information

Below is the link to the electronic supplementary material.

Supplementary Material 1

## Data Availability

The datasets used and analyzed during the current study are available from the corresponding author on reasonable request.

## Abbreviations

vEDS: vascular Ehlers-Danlos syndrome
PH: Pulmonary hemorrhage
P/HTX: Pneumothorax/hemothorax
B/E: Bullae/emphysema
EDS: Ehlers-Danlos syndromes
WBC: White blood cell
CRP: C reactive protein
ESR: Erythrocyte sedimentation
ANA: Antinuclear antibody
ANCA: Anti-neutrophil cytoplasmic antibody
VATS: Video-assisted thoracic surgery
GGO: Ground glass opacities
SD: Standard deviation
IQR: Interquartile range
