# Supplementary material for: Pulmonary hemorrhage as an early clue: an integrated clinical-imaging-genetic diagnostic insight for vascular Ehlers-Danlos syndrome
Source: Orphanet J Rare Dis. 2026 Mar 23;21:174. doi: 10.1186/s13023-026-04327-0 (PMC13134318; doi:10.1186/s13023-026-04327-0)
Supplement: Supplementary file 1 — Supplementary Material 1 [file 13023_2026_4327_MOESM1_ESM.pdf]

Supplementary Table S1. Definitions of chest CT abnormalities.

	**Definition**
**Nodule**	A nodule is a circumscribed, typically round opacity, less than or equal to 30 mm in average diameter. A rounded lesion larger than 30 mm is referred to as a mass.
**Cavity**	A cavity is an abnormal gas- or fluid-filled structure with a typically thick and often irregular wall, usually produced by the expulsion or drainage of a necrotic part of the lesion via the bronchial tree.
**Halo**	A halo is a rim of ground-glass opacity surrounding a nodule, mass, or consolidation.
**Lung bullae**	A bulla is a circumscribed air-containing cystic structure in the lung parenchyma lined by a thin layer of collapsed lung parenchyma.
**Emphysema**	Emphysema is characterized by irreversible enlarged airspaces distal to and originating from the terminal bronchioles, with destruction of alveolar walls.
**Ground-glass opacity**	Ground-glass refers to an area of increased attenuation that does not completely obscure the underlying bronchial and vascular structures.
**Bronchiectasis**	Bronchiectasis indicates a clinical condition of irreversible bronchial dilatation.

Supplementary Table S2. Genetic mutation sites and pathogenicity of vEDS patients with initial respiratory symptoms.

**Patient**	**Nucleotide change**	**Amino acid change**	**ACMG Guideline**	
			**Class**	**Evidence**
Patient 1	c.3149G>A	p.Gly1050Asp	Likely pathogenic	PP3+PM2+PM5+PM1+PP2+PP5
Patient 2	c.3229G>A	p.Gly1077Ser	Pathogenic	PS4+PP3+PM2+PM5+PM1+PP2+PP5

Patient 3	c.2383G>C	p.Gly795Arg	Pathogenic	PP3+PM2+PM5+PP2 +PP5
Patient 4	c.2393G>T	p.Gly798Val	Likely pathogenic	PP3+PM2+PM5+PP2
Patient 5	c.1816-2A>G	Splicing	Likely pathogenic	PVS1+PM2+PP5
Patient 6	c.690+1G>T	Splicing	Likely pathogenic	PVS1+PM2
Patient 7	c.1142G>A	p.Gly381Asp	Pathogenic	PS4+PP3+PM2+PM1 +PP2+PP5
Patient 8	c.1897G>A; c.3064C>T	p.Gly633Arg; p.Pro1022Ser	Pathogenic;VUS	PP3+PM2+PS4+PM5 +PP2+PP5; PM1+PM2+PP2
Patient 9	EX42-EX43 Del	Indel	Likely pathogenic	
Patient 10	c.2672G>T	p.Gly891Val	Likely pathogenic	PP3+PM2+PM5+PM1 +PP2+PP5
Patient 11	c.3256-43T>G	Splicing	Likely pathogenic	PS4+PM2+PP5
Patient 12	c.3212G>T	p.Gly1071Val	Pathogenic	PS4+PP3+PM2+PM1 +PP2+PP5
Patient 13	c.1697C>T	p.Pro566Leu	Likely benign	PP2+BP6+BS1
Patient 14	c.2283+2T>G	Splicing	Likely pathogenic	
Patient 15	c.3562G>A	p.Gly1188Arg	Pathogenic	PS4+PP3+PM2+PM5 +PM1+PP2+PP5
Patient 16	c.2024G>T	p.Gly675Val	Pathogenic	PS4+PP3+PM2+PM5 +PM1+PP2+PP5
Patient 17	c.547G>A	p.Gly183Ser	Pathogenic	PS4+PP3+PM2+PM5 +PM1+PP2+PS3+PP5
Patient 18	c.2022+1G>A	Splicing	Likely pathogenic	PVS1+PM2
Patient 19	c.3061C>A	p.Leu1021Ile	Benign	PP2+BP6+BV1
Patient 20	c.2897G>A	p.Gly966Asp	Likely pathogenic	PP3+PM2+PM1+PM5 +PP2
Patient 21	c.3256G>C	p.Gly1086Arg	Likely pathogenic	PP3+PM2+PM5+PP2

Patient 22	c.2445+1delG	Splicing	Likely pathogenic	PVS1+PM2
Patient 23	c.983G>C	p.Ser328Thr	VUS	PM2+PM1+PP2
Patient 24	c.1869+1G>T	Splicing	Likely pathogenic	PVS1+PM2
Patient 25	c.3329G>A	p.Gly1110Glu	Pathogenic	PS4+PP3+PM2+PM1 +PP2+PP5
Patient 26	c.4072C>T	p.Arg1358*	Pathogenic	PVS1+PM2+PS4+PP5

*The gene mutation results for the remaining six patients were unavailable for retrieval; however, their electronic medical records clearly documented *COL3A1* gene mutations, all of which were classified as pathogenic or likely pathogenic.

Supplementary Table S3. Characteristics of ten patients with vEDS who presented with pulmonary hemorrhage.

	Patient 1	Patient 2	Patient 3	Patient 4	Patient 5	Patient 6	Patient 7	Patient 8	Patient 9	Patient 10
**Gender**	M	M	M	F	M	M	F	F	F	M
**Age**	16	30	23	22	17	17	14	19	17	16
**Respiratory manifestation**										
Chest pain	-	+	+	+	+	+	+	+	-	+
Pneumothorax	+	-	+	-	+	+	+	-	+	+
**Other manifestation**										
Joint hypermobility	+	+	-	+	+	+	-	+	-	-
Thin and translucent skin	-	-	-	+	-	+	-	-	-	-
Ecchymosis	+	-	-	-	-	+	-	+	+	-
**Initial chest CT finding**										
Nodule	-	-	+	+	-	+	+	+	+	+

Cavity	-	+	+	+	-	+	-	+	-	+
GGO	+	-	-	-	+	+	+	+	+	+
Lung bullae	-	-	-	-	-	-	-	-	-	-
**Pathogen examination**	NA	-	-	-	NA	-	-	NA	-	NA
**Procedure**										
Bronchoscopy	+	+	+	+	-	+	+	-	+	-
Lung biopsy										
Percutaneous	-	-	+	-	-	-	-	-	+	-
Transbronchial	-	+	-	+	-	-	-	-	-	-
Surgical	-	-	+	+	-	-	-	-	-	-
Closed thoracic drainage	+	-	-	-	+	+	-	-	+	+
**Treatment**										
Antibiotic	+	+	+	+	+	+	+	-	-	-
Anti-tuberculous	-	+	+	-	-	-	-	-	-	-

**Diagnostic duration*, years**	10.78	4.57	3.00	2.07	0.41	0.21	0.59	0.04	0.09	0.10
**Family history**	+	+	-	+	+	-	-	+	-	-

GGO: Ground-glass opacity; NA: Not available

* Diagnostic duration: Time interval between first visit and diagnosis.

Supplementary Table S4. Demographics and clinical features of patients with vEDS who visited the hospital before and after 2022.

	First visit before 2022 (n=11)	First visit after 2022 (n=21)	*P*
**Age of first visit, years**	24.3±7.09	23.5±11.1	0.84
**Male, n (%)**	10 (90.9)	14 (66.7)	0.28
**Common features**	8 (72.7)	12 (57.1)	0.63
**Laboratory test**			
WBC, $\times 10^9$/L	6.39 [5.50, 9.88]	6.08 [5.62, 8.76]	0.80
Neutrophil, $\times 10^9$/L	3.93 [3.44, 4.77]	4.19 [3.60, 5.61]	0.57
Neutrophil, %	57.29 [56.1, 61.5]	63.4 [55.8, 68.1]	0.32
C reactive protein, mg/L	2.44 [0.90, 3.86]	3.47 [0.74, 8.66]	0.55
ESR, mm/h	5.00 [2.75, 5.25]	7.00 [6.00, 12.0]	0.02
Positive ANA, %	0 (0.00)	1 (7.14)	1

Supplementary Table S5. Comparison of the 2017 international Ehler-Danlos syndrome classification and our experience-based insights.

Patient ID	Year of first visit	2017 Diagnostic Criteria		Empirical summary			Diagnosis Duration (years)	Misdiagnosis	Invasive Procedures for Diagnosis	Nucleotide change
		Major clinical findings	Other clinical findings	Hemoptysis	Hemoptysis related imaging features	Other respiratory manifestation				
Patient 1	2012	Family history	Common signs; pneumothorax	√	√	Pneumothorax	10.78	√	√	c.3149G>A
Patient 2	2016	Family history	Common signs	√	√	×	4.57	√	√	c.3229G>A
Patient 3	2018	×	Pneumothorax	√	√	Pneumothorax	3.00	√	√	c.2383G>C
Patient 4	2019	Family history	Common signs	√	√	×	2.07	√	√	c.2393G>T
Patient 5	2021	Family history	Common signs; pneumothorax	√	√	Pneumothorax	0.41	√	√	c.1816-2A>G
Patient 6	2021	Arterial aneurysm*	Common signs; pneumothorax	√	√	Pneumothorax	0.21	√	√	c.690+1G>T
Patient 7	2023	×	Pneumothorax	√	√	Pneumothorax	0.59	√	√	c.1142G>A

**Patient 8**	**2024**	**Family history**	**Common sign**	√	√	×	**0.04**	×	×	**c.1897G>A; c.3064C>T**
**Patient 9**	**2024**	×	**Common signs; pneumothorax**	√	√	**Pneumothorax**	**0.09**	×	×	**EX42-EX43 Del**
**Patient 10**	**2025**	×	**Pneumothorax**	√	√	**Pneumothorax**	**0.10**	×	×	**c.2672G>T**
**Patient 11**	**2021**	×	**Pneumothorax**	√	×	**Pneumothorax**	**before first visit**	×	×	**c.3256-43T>G**
**Patient 12**	**2023**	×	**Common signs; pneumothorax**	√	×	**Pneumothorax**	**before first visit**	×	×	**c.3212G>T**
**Patient 13**	**2023**	**Family history; arterial rupture**	**Common signs**	√	×	×	**0.27**	×	×	**c.1697C>T**
**Patient 14**	**2016**	**sigmoid colon rupture**	**Common signs; hemothorax**	×	√	**Hemothorax**	**6.44**	√	×	**c.2283+2T>G**
**Patient 15**	**2021**	×	**Common signs**	×	×	×	**before first visit**	×	×	**c.3562G>A**

**Patient 16**	**2021**	**Family history; arterial dissection**	×	×	×	×	**0.24**	√	×	**c.2024G>T**
**Patient 17**	**2022**	**Family history; Arterial aneurysms, and dissection**	**Common signs; pneumothorax**	×	×	**Pneumothorax**	**0.19**	×	×	**c.547G>A**
**Patient 18**	**2022**	**Arterial aneurysms**	×	×	×	×	**0.64**	×	×	**c.2022+1G>A**
**Patient 19**	**2022**	×	**Common signs**	×	×	×	**before first visit**	×	×	**c.3061C>A**
**Patient 20**	**2024**	**Family history**	**Common signs; pneumothorax**	×	√	**Pneumothorax**	**0.12**	×	×	**c.2897G>A**
**Patient 21**	**2024**	**Family history**	**Pneumothorax**	×	×	**Pneumothorax**	**before first visit**	×	×	**c.3256G>C**
**Patient 22**	**2024**	**Arterial aneurysms**	**Common signs; pneumothorax**	×	√	**Pneumothorax**	**0.11**	×	×	**c.2445+1delG**
**Patient 23**	**2024**	**Family history**	**Pneumothorax**	×	×	**Pneumothorax**	**0.14**	×	×	**c.983T>C**

**Patient 24**	**2024**	×	**Common signs; pneumothorax**	×	×	**Pneumothorax**	**0.62**	×	×	**c.1869+1G>T**
**Patient 25**	**2024**	**Arterial aneurysms, and dissection**	×	×	×	×	**0.09**	×	×	**c.3329G>A**
**Patient 26**	**2024**	×	**Common signs**	×	×	×	**before first visit**	×	×	**c.4072C>T**
**Patient 27**	**2022**	×	**Pneumothorax**	√	×	**Pneumothorax**	**before first visit**	×	×	
**Patient 28**	**2019**	**Arterial dissection**	**Common signs**	×	×	×	**1.92**	√	×	
**Patient 29**	**2023**	**Arterial aneurysms, and dissection**	×	×	×	×	**0.09**	×	×	
**Patient 30**	**2023**	**Family history; arterial aneurysms**	**Common signs; pneumothorax**	×	×	**Hemothorax**	**0.19**	×	×	

**Patient 31**	**2024**	**Family history; Arterial aneurysms, dissection and rupture**	**Common signs**	×	×	×	**0.15**	√	×	
**Patient 32**	**2024**	**Family history; arterial aneurysms**	×	×	×	×	**0.18**	×	×	

*** An aneurysm was found in Patient 6 during the follow-up after diagnosis**

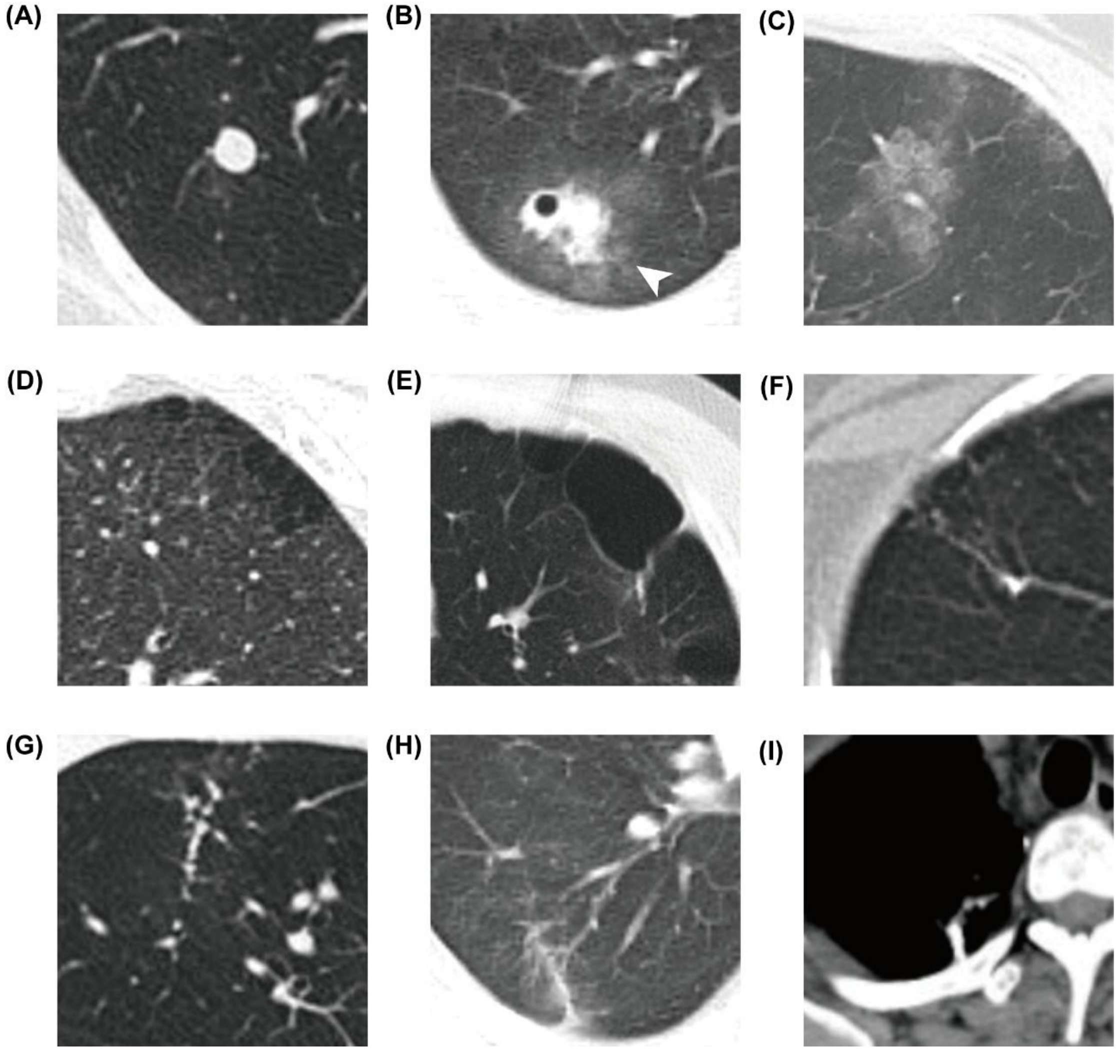

Supplementary Fig. S1. Representative chest CT abnormalities of vascular Ehlers-Danlos syndrome. (A) Solid nodule. (B) Cavitary nodule with halo sign (indicated by the white arrow). (C) Ground-glass opacities. (D) Emphysema. (E) Bullae. (F) Bronchiolectasis. (G) Fibrous nodules. (H) Linear opacity. (I) Calcified nodule.
